# A nomogram model for predicting the risk factors of plastic bronchitis in children

**DOI:** 10.3389/fmed.2026.1794234

**Published:** 2026-04-10

**Authors:** Qiao Zhang, Zhihua Zhao, Ting Yang, Jianchuan Chen, Jihong Dai

**Affiliations:** Department of Respiratory Medicine, Children’s Hospital of Chongqing Medical University, National Clinical Research Center for Children and Adolescents’ Health and Diseases, Ministry of Education Key Laboratory of Child Development and Disorders, Chongqing Key Laboratory of Child Rare Diseases in Infection and Immunity, Chongqing, China

**Keywords:** bronchoscopy, nomogram model, plastic bronchitis, prediction model, risk factors

## Abstract

**Objective:**

Plastic bronchitis (PB) in children is a critical condition requiring prompt intervention. Early identification of high-risk patients is crucial for timely bronchoscopic management. This study aimed to develop and validate a novel nomogram model for the early prediction of PB risk in pediatric patients.

**Methods:**

We conducted a retrospective analysis of clinical data from children with respiratory conditions. The cohort was divided into a training set (*n* = 326) and an independent validation set (*n* = 136). Univariate and multivariate logistic regression analyses were performed to identify independent risk factors for PB by comparing demographics, clinical symptoms, laboratory findings, and imaging features between groups. A predictive nomogram was subsequently constructed based on the results of the multivariate analysis.

**Results:**

Multivariate analysis identified seven independent predictors of PB: elder age, longer cough duration, *mycoplasma pneumoniae* infection, atelectasis, lung consolidation, pleural effusion, and pleurisy. The nomogram demonstrated excellent discriminative ability, with an area under the receiver operating characteristic curve (AUC) of 0.920 in the training set and 0.929 in the validation set. Good calibration was confirmed by the Hosmer–Lemeshow test (*p* = 0.545).

**Conclusion:**

We successfully developed and validated a practical nomogram incorporating seven readily available clinical parameters. This model serves as a reliable and non-invasive tool for the early assessment of PB risk in children, potentially facilitating timely clinical decision-making and intervention.

## 1 Introduction

The global COVID-19 pandemic has altered the epidemiological landscape of numerous respiratory pathogens. Prior to its emergence, *Mycoplasma pneumoniae* was recognized as the predominant infectious trigger of plastic bronchitis (PB) in children. However, recent clinical observations indicate a shift, with an increasing variety of pathogens—including respiratory syncytial virus (RSV), *Chlamydia pneumoniae*, adenovirus, and influenza virus—being implicated in PB onset (1, 2). This evolving etiology underscores the need to revisit the risk profile and predictive tools for this condition.

Plastic bronchitis is a rare but severe respiratory syndrome characterized by the formation of extensive, branching fibrinopurulent casts within the bronchial tree, leading to acute or subacute airway obstruction. Patients typically present with non-specific symptoms such as persistent cough, dyspnea, fever, and pleuritic chest pain. Due to its rarity and symptomatic overlap with more common respiratory illnesses, PB is often under-recognized, and its true prevalence remains unclear. Nevertheless, its potential to progress rapidly to life-threatening complications, including respiratory failure, necessitates heightened clinical vigilance (3, 4).

The diagnosis of PB hinges on a high index of suspicion, integrating clinical presentation, characteristic imaging findings (e.g., patchy consolidations and atelectasis), and ultimately, confirmation via bronchoscopy, which reveals the pathognomonic casts (5). Crucially, bronchoscopy with bronchoalveolar lavage (BAL) serves not only as the diagnostic gold standard but also as the primary therapeutic intervention by mechanically clearing the obstructing casts. Consequently, the early and accurate identification of children at high risk for PB is paramount to guiding timely bronchoscopic evaluation and management, thereby improving outcomes.

Existing efforts to stratify PB risk are limited. To date, only two published studies have proposed clinical prediction models, both developed prior to the COVID-19 pandemic and exclusively focused on PB secondary to refractory *Mycoplasma pneumoniae* pneumonia (6, 7). These models, while valuable, may not fully capture the current, more diverse pathogenic spectrum, limiting their generalizability and contemporary clinical applicability.

To address this gap, we conducted a study encompassing a broader range of etiologies, including *M. pneumoniae*, RSV, adenovirus, influenza, and other bacterial infections. Our objective was to develop and validate a novel, clinically practical nomogram. This model integrates readily available parameters from clinical presentation, routine laboratory tests, and radiological examinations to assist clinicians in the early identification of pediatric patients at high risk for PB, facilitating prompt and potentially life-saving intervention.

## 2 Materials and methods

### 2.1 Patients

This retrospective study aimed to develop a predictive model for plastic bronchitis (PB) in children with severe pneumonia. We enrolled pediatric patients who were admitted to the Respiratory Department of the Children’s Hospital of Chongqing Medical University between January 2020 and March 2025. This post-pandemic period was intentionally selected to capture the contemporary etiological spectrum of severe respiratory infections.

Children were eligible if they met the diagnostic criteria for severe pneumonia, defined as follows: (1) acute respiratory symptoms, including fever, cough, or wheezing; with or without auscultation abnormalities, such as decreased bubbles or breath sounds; (2) persistent fever or continued progression of clinical symptoms and chest radiographic signs; (3) consolidation of the lung or atelectasis. The exclusion criteria were: combined with tuberculosis, cardiovascular disease, liver and kidney disease, or immunodeficiency. Each child had at least one guardian who gave written informed consent before participating in the study.

Combined with clinical manifestation and chest imaging examination, bronchoscopy should be considered when lobar consolidation or atelectasis existed or clinical manifestation were not improved or progresesed after antibiotics treatment.

### 2.2 Groups

The hospital has two geographically distinct branches, each staffed by a largely fixed, independent team of clinicians, nurses, laboratory technicians, and radiologists. Patients admitted to the larger, main Branch A were allocated to the training set for model development. Conversely, patients from Branch B constituted the independent validation set, used solely for testing the model’s performance on a distinct patient population managed by a separate clinical team.

The definitive classification of patients into Plastic Bronchitis (PB) or non-PB groups was based solely on bronchoscopic findings, serving as the gold standard. PB Group: Inclusion required the direct visualization and retrieval of characteristic bronchial casts. These casts were defined as cohesive, branching, fibrin-rich formations partially obstructing the bronchial lumen. Upon extraction via lavage, suction, or biopsy forceps, they exhibited the pathognomonic property of expanding into an arborizing structure when immersed in saline. Non-PB Group: This group comprised patients in whom bronchoscopy revealed no evidence of such casts. Findings were limited to common inflammatory changes such as mucosal congestion, edema, the presence of rales, or flocculent secretions.

### 2.3 Data collection

This is a retrospective study that we collected data through the electronic medical record system. The following general clinical characteristics were collected: sex, age, duration of fever, cough and wheeze status, presence of mixed infection, extrapulmonary organ involvement (including liver dysfunction, renal dysfunction, abnormal myocardial dial zymogram, and abnormal neurological symptoms), Pulmonary Imaging Examination revealed the following changes: consolidation of the lung, atelectasis, bronchiectasia, pleural effusion and pleurisy. The following laboratory analysis data were obtained: white blood cells (WBC), c-reactive protein (CRP), lactate dehydrogenase (LDH) and procalcitonin (PCT).

### 2.4 Statistical analysis

All statistical analyses in this study were performed using R software (version x.y.z; https://www.r-project.org/). Continuous variables are presented as mean ± standard deviation and were compared using the Student’s *t*-test. Categorical variables are presented as numbers (percentages) and were compared using the Chi-squared test. A two-sided *p*-value <0.05 was considered statistically significant.

To develop the prediction model, univariable and multivariable logistic regression analyses were performed using the glm function. Variables with significant associations in the univariable analysis (*p* < 0.05) were included in the multivariable model to identify independent risk factors. Based on the final multivariable model, a nomogram was constructed using the rms package.

The model’s performance was evaluated in both the training and validation sets. Discriminative ability was assessed by the area under the receiver operating characteristic curve (AUC), calculated using the pROC package. Calibration, which measures the agreement between predicted probabilities and observed outcomes, was evaluated using a calibration plot and the Hosmer–Lemeshow test via the rms package. Furthermore, the clinical utility of the nomogram was quantified using decision curve analysis (DCA) implemented with the rmda package.

## 3 Results

**Figure 1:**
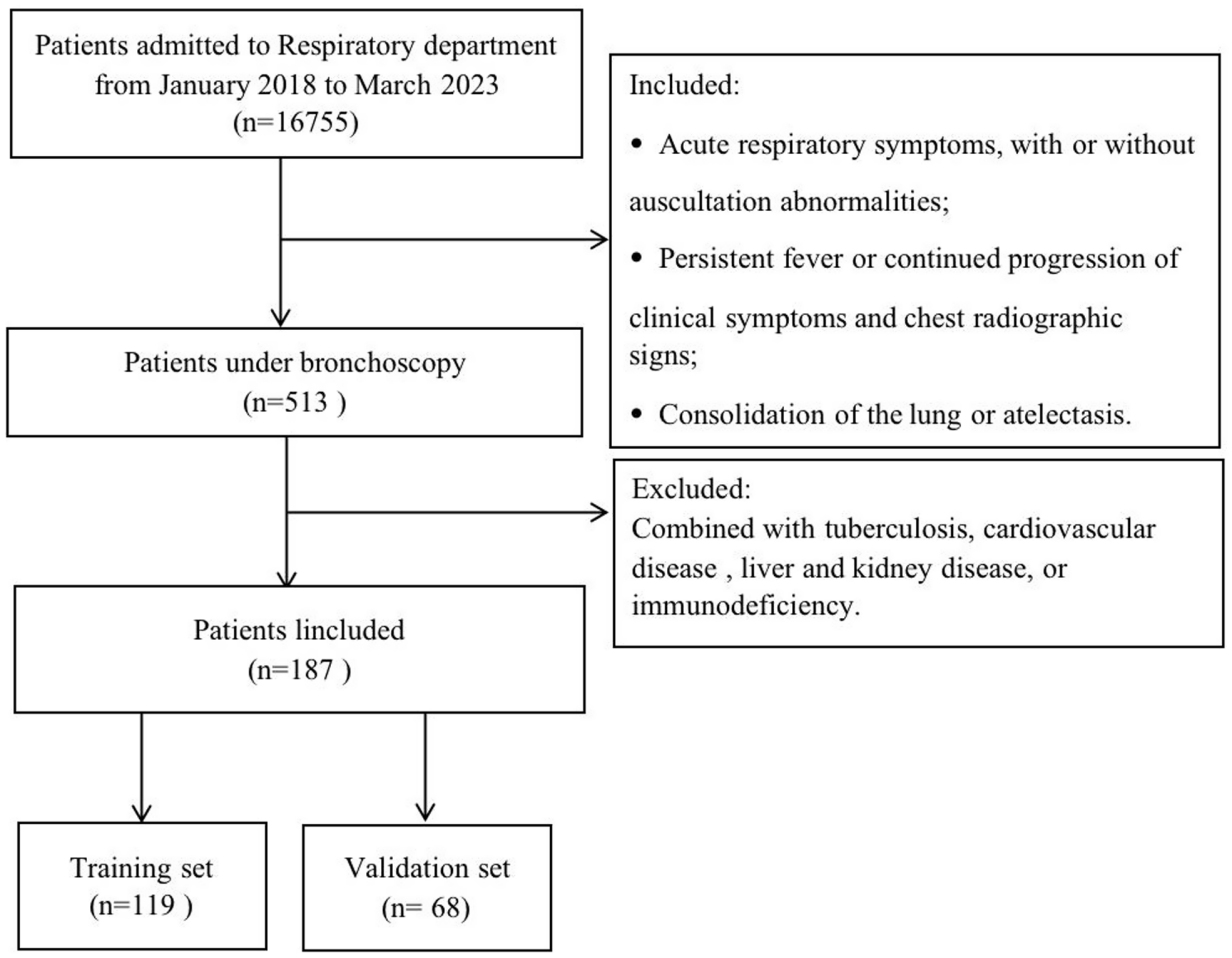
The flow diagram.

Of the 16,755 children admitted to the Respiratory Department of the Children’s Hospital of Chongqing Medical University between January 2020 and March 2025, bronchoscopy was performed on 927 patients with large consolidation lesions. After excluding patients with other systemic complications, 462 children were ultimately included in the analysis, with 326 assigned to the training set and 136 to the validation set. The patient selection process is summarized in the flowchart (Figure 1).

### 3.1 Characteristics of the study cohort

The baseline clinical characteristics, laboratory results, and imaging findings were well-balanced between the training and validation sets, with no statistically significant differences observed (*p* > 0.05).

In the training set, male and female patients accounted for 58.8 and 41.2%, respectively, whereas the validation set comprised equal proportions of both sexes (50.0% each). The mean age was 54.4 ± 42.7 months in the training set and 38.8 ± 30.2 months in the validation set. Regarding clinical symptoms, the mean duration of fever was 6.4 ± 6.3 days in the training set versus 4.7 ± 4.4 days in the validation set; the mean duration of cough was 14.0 ± 9.1 days and 14.5 ± 8.5 days, respectively. Wheezing was present in 40.3% of patients in the training set and 55.9% in the validation set.

The detected pathogens in the training set were *Streptococcus pneumoniae* (18.5%), influenza virus (10.1%), *Mycoplasma pneumoniae* (35.3%), adenovirus (16.0%), parainfluenza virus (10.1%), Staphylococcus (5.9%), respiratory syncytial virus (10.9%), and *Haemophilus influenzae* (12.6%). Corresponding proportions in the validation set were 32.4, 8.8, 30.9, 13.2, 8.8, 2.9, 2.9, and 13.2%.

No significant inter-set differences were noted in routine laboratory parameters. On imaging, lung consolidation was observed in 11.8% (training set) versus 2.9% (validation set), atelectasis in 33.6% versus 22.1%, pleural effusion in 14.3% versus 10.3%, bronchiectasis in 4.2% versus 2.9%, and pleurisy in 10.9% versus 8.8%.

The prevalence of plastic bronchitis was 60.5% in the training set and 26.5% in the validation set. All detailed baseline data are summarized in Table 1.

**Table 1:** Characteristics of the training set and validation set.

Characteristics	Training set (*n* = 326)	Validation set (*n* = 136)	*p*-value
Sex			
Male	192 (58.8)	68 (50.0)	0.285
Female	134 (41.2)	68 (50.0)	
Age (month)	54.366 ± 42.726	38.809 ± 30.249	0.052
Fever	6.394 ± 6.340	4.693 ± 4.350	0.069
Cough	13.990 ± 9.062	14.500 ± 8.477	0.546
Wheeze	131 (40.3)	76 (55.9)	0.052
*Streptococcus pneumoniae*	60 (18.5)	44 (32.4)	0.052
Influenza virus	33 (10.1)	12 (8.8)	1.000
*Mycoplasma pneumoniae*	115 (35.3)	42 (30.9)	0.630
Adeno virus	52(16.0)	18 (13.2)	0.675
Parainfluenza virus	33 (10.1)	12 (8.8)	1.000
*Staphylococcus aureus*	19(5.9)	4 (2.9)	0.491
Respiratory syncytial virus	36 (10.9)	4 (2.9)	0.090
*Haemophilus influenzae*	41 (12.6)	18 (13.2)	1.000
WBC	11.637 ± 6.048	11.014 ± 5.563	0.490
CRP	37.457 ± 29.692	23.434 ± 20.370	0.063
PCT	17.500 ± 2.980	4.116 ± 0.967	0.885
LDH	546.033 ± 503.442	423.924 ± 348.582	0.221
Extrapulmonary	90 (27.7)	40 (29.4)	0.867
Lung consolidation	38 (11.8)	4 (2.9)	0.055
Atelectasis	110 (33.6)	30 (22.1)	0.133
Pleural effusion	47 (14.3)	14 (10.3)	0.501
Bronchiectasia	14 (4.2)	4 (2.9)	1.000
Pleurisy	36 (10.9)	12 (8.8)	0.803
Plastic bronchitis	197 (60.5)	36 (26.5)	0.052

### 3.2 Independent predictors in the training set

**Figure 2:**
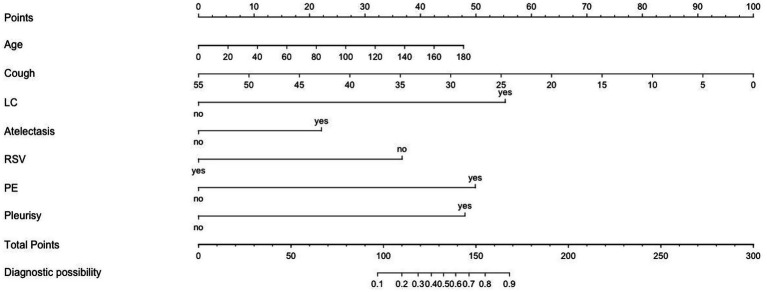
Nomogram to predict the risk factors of PB.

To identify independent predictors of plastic bronchitis, we first performed univariable analysis (Mann–Whitney *U* test) on the candidate variables presented in Table 1, which preliminarily screened out ten variables with significant associations (Figure 2). To account for potential collinearity among these variables, they were subsequently entered into a multivariable logistic regression model. This analysis confirmed seven variables as independent predictors significantly associated with PB in the training set: elder age, longer cough duration, the presence of atelectasis, lung consolidation, *Mycoplasma pneumoniae* infection, pleural effusion, and pleurisy (Table 2).

**Table 2:** Univariable analysis and multivariate logistic regression analysis for risk factors of PB.

Characteristics	Univariable logistic analysis		Multivariate logistic regression	
	Odds ratio (95% CI)	*p*	Odds ratio (95% CI)	*p*
Adv	0.88–9.13 (2.83)	0.082	—	—
Age	1.02–1.05 (1.03)	0	1–1.04 (1.02)	0.023
Cough	0.91–0.99 (0.95)	0.023	0.82–0.96 (1.02)	0.003
CRP	1.02–1.07 (1.05)	0.002	0.99–1.06 (1.02)	0.149
Extrapulmonary	0.52–2.75 (1.2)	0.665	—	—
Atelectasis	1.91–12.22 (4.84)	0.001	1.07–16.8 (4.24)	0.04
Fever	1.05–1.23 (1.14)	0.001	—	—
Lung consolidation	1.28–80.3 (10.14)	0.028	1.72–406.67 (26.46)	0.019
*Haemophilus influenzae*	0.06–0.65 (0.19)	0.008	0.04–1.63 (0.25)	0.147
Influenza virus	0.54–8.18 (2.1)	0.287	—	—
LDH	1-1 (1)	0.057	—	—
RSV	1.51–8.46 (3.57)	0.004	0.06–1.24 (0.28)	0.093
Parainfluenza virus	0.19–2.06 (0.62)	0.436	—	—
PCT	0.93–1.39 (1.13)	0.224	—	—
MP	0.04–0.62 (0.16)	0.008	0.01–0.86 (0.1)	0.036
Sex	0.5–2.22 (1.05)	0.893	—	—
*Staphylococcus aureus*	0.49–35.9 (4.18)	0.192	—	—
*Streptococcus pneumoniae*	0.23–1.5 (0.59)	0.267	—	—
WBC	0.93–1.05 (0.99)	0.77	—	—
Wheeze	0.08–0.42 (0.19)	0	—	—
Pleurisy	1.15–73.32 (9.2)	0.036	1.15–196.87 (15.07)	0.039
Pleural effusion	1.68–102.91 (13.14)	0.014	1.12–157.85 (13.31)	0.04
Bronchiectasia	0-0 (0)	0.988	—	—

### 3.3 Establishment of the nomogram

A nomogram was developed to predict the probability of PB based on seven predictors: age, sex, atelectasis, lung consolidation, *Mycoplasma pneumoniae* infection, pleurisy, and pleural effusion (Figure 2). This nomogram provides a graphical representation of the multivariable logistic regression model. To use it, a point score is assigned for each predictor according to its corresponding scale at the top axis. The sum of these individual scores yields a total points value, which can then be projected downward to the bottom axis to read the estimated individual probability of PB.

### 3.4 Validation and calibration of the nomogram

**Figure 3:**
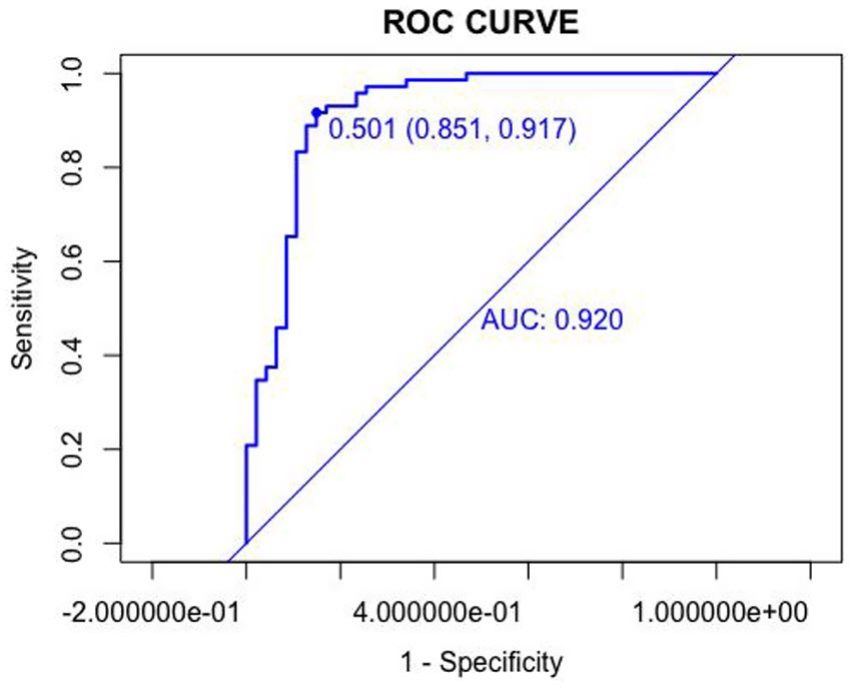
Receiver operating characteristic (ROC) curves in the training set.

**Figure 4:**
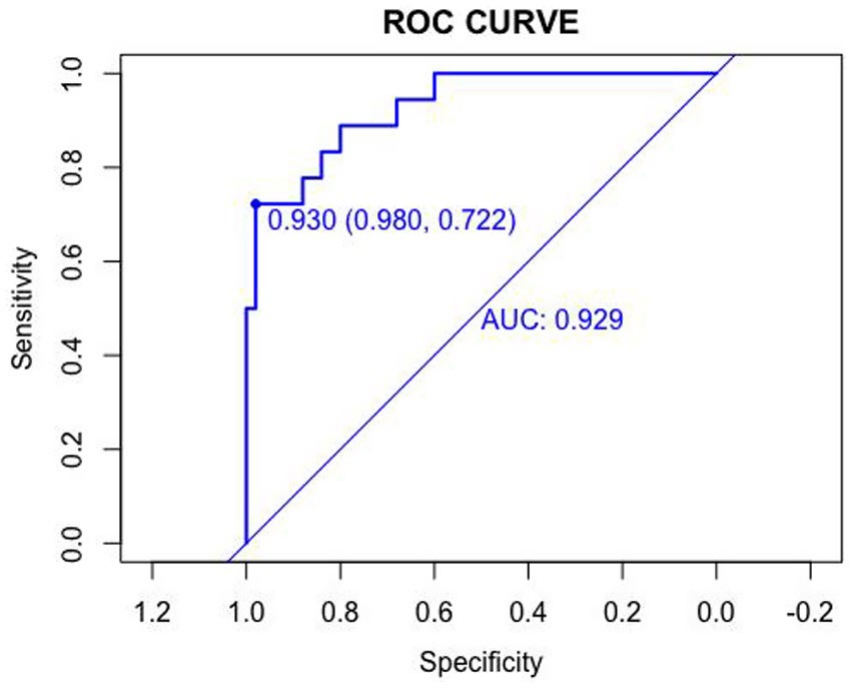
Receiver operating characteristic (ROC) curves in the validation set.

The discriminative ability of the nomogram was assessed using receiver operating characteristic (ROC) curves. The area under the ROC curve (AUC) was 0.920 in the training set and 0.929 in the validation set, indicating excellent discrimination. The optimal cutoff values were 0.501 (sensitivity: 0.851, specificity: 0.917) in the training set and 0.930 (sensitivity: 0.980, specificity: 0.722) in the validation set (Figures 3, 4).

**Figure 5:**
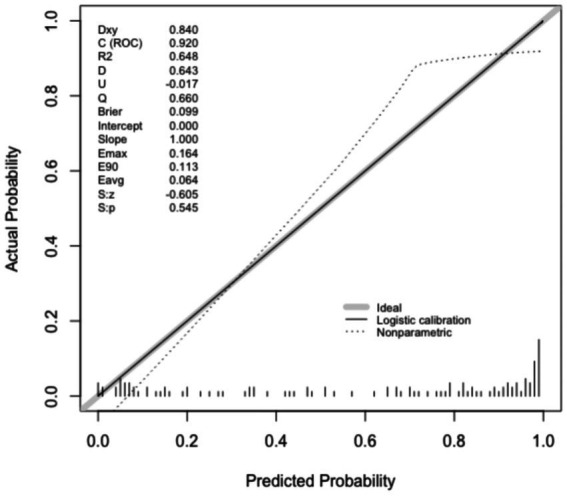
Calibration curves in the training set.

**Figure 6:**
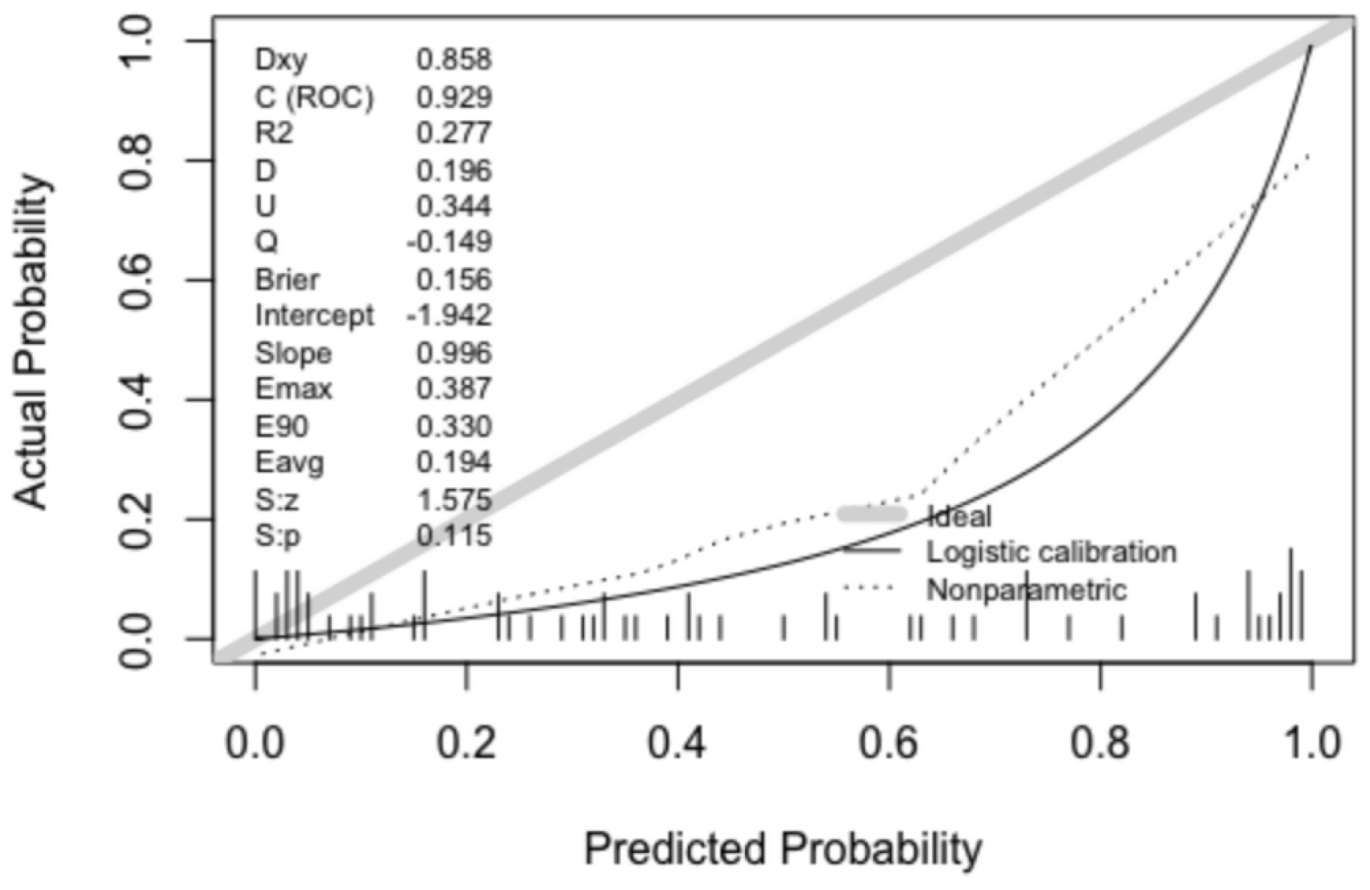
Calibration curves in the validation set.

Calibration of the nomogram was evaluated with calibration plots and the Hosmer–Lemeshow test. As shown in Figures 5, 6, the calibration curves demonstrated good agreement between the predicted probabilities and the actual observed outcomes for PB in both the training set (*p* = 0.545) and the validation set (*p* = 0.115). A *p*-value greater than 0.05 suggests that the model is well-calibrated.

**Figure 7:**
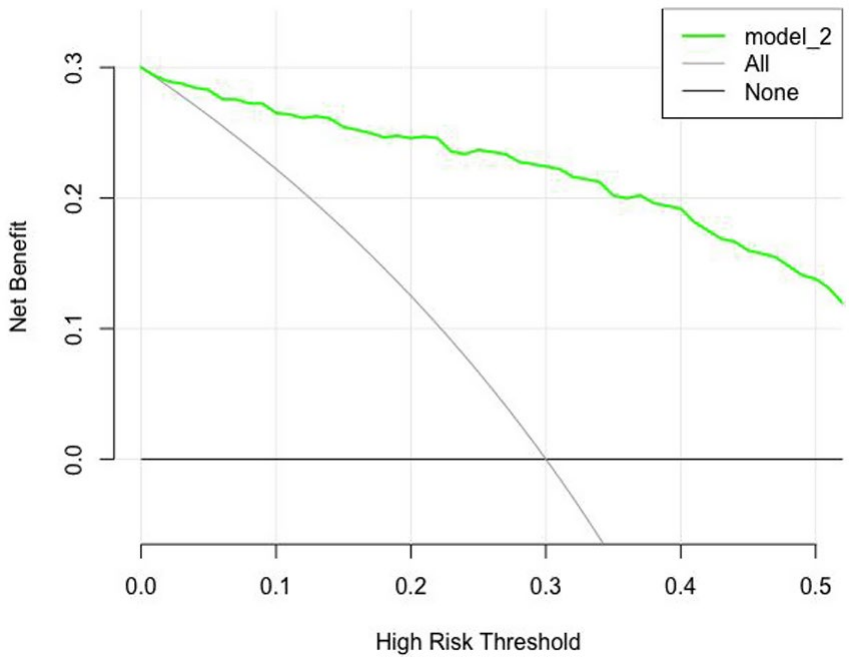
Decision curve analyses in the training set.

**Figure 8:**
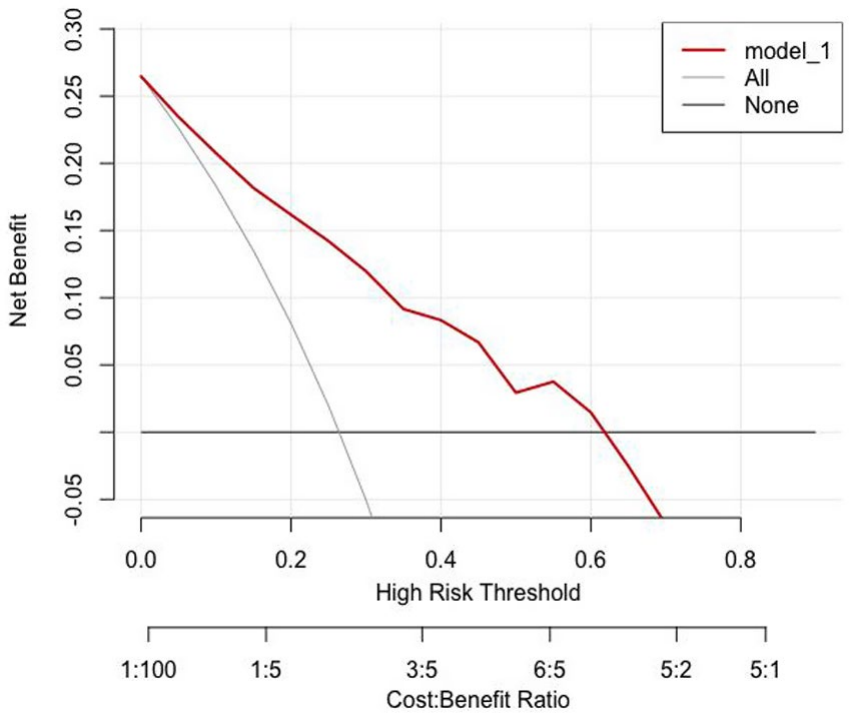
Decision curve analyses in the validation set.

The clinical utility of the prediction model was quantified using decision curve analysis (DCA). The analysis revealed that across a wide range of threshold probabilities, the use of the nomogram to predict PB provided a higher net benefit compared to the strategy of treating all patients or treating none (Figures 7, 8).

## 4 Discussion

Plastic bronchitis remains a rare yet life-threatening respiratory syndrome in children, characterized by the formation of obstructive bronchial casts that lead to acute airway obstruction, respiratory distress, and, in severe cases, fatal outcomes (5, 6). Its etiology is multifaceted and not fully elucidated, with reported associations spanning congenital heart disease post-surgery (7–9), asthma (10), bronchial thermoplasty (11), heart failure (12), lung transplantation (13), and notably, acute respiratory infections (14). The pathogenesis may involve increased pulmonary venous pressure and lymphatic leakage in cardiac cases (15–17), or excessive inflammatory secretion from bronchial epithelium triggered by infections (3, 14, 18). Despite a reported incidence of approximately 6.8 per 100,000 and mortality around 7% (19), recent epidemiological trends suggest a gradual rise in cases, potentially reflecting regional characteristics in Asia (19, 20). Clinically, PB poses a significant diagnostic challenge due to its non-specific presentation—commonly fever, cough, and consolidation on imaging—which overlaps with severe pneumonia, often necessitating bronchoscopy for definitive diagnosis and urgent intervention (21). The underlying mechanisms are thought to involve dysregulated immune responses and possibly antibiotic resistance, yet a unified pathological understanding is still lacking (22).

In our cohort, several distinct clinical patterns emerged. While the duration of fever was shorter in PB patients, fever itself was more prevalent, suggesting a pronounced but possibly more acute inflammatory phase. The earlier time to bronchoscopy and prompt defervescence post-procedure in the PB group underscore the critical role of timely cast removal in alleviating obstruction and inflammation (23, 24). The shorter duration of cough in PB children raises intriguing questions—whether it results from a suppressed cough reflex impairing clearance or from rapid cast formation that physically limits effective coughing. The significantly higher incidence of extrapulmonary complications, pleural effusion, and elevated LDH in the PB group indicates a more intense local and systemic inflammatory response. Radiologically, the predominance of homogeneous consolidation confined to single lung segments, rather than diffuse multi-segmental exudates, may offer a clue to the focal, obstructive nature of cast formation. Pleural effusion likely arises from severe local inflammation and increased hydrostatic pressure secondary to airway occlusion.

Contrary to some previous reports describing fulminant viral-induced PB, most cases in our post-pandemic cohort were clinically moderate. This may reflect earlier detection and intervention or a different pathophysiology in the current pathogen landscape. Importantly, we observed that residual or recurrent casts were found in some children even on repeat bronchoscopy, highlighting the potential for persistent or recurrent disease and the need for complete airway clearance.

A key limitation of the existing literature is the reliance on univariable analyses or models derived predominantly from *Mycoplasma pneumoniae*-associated PB in the pre-COVID-19 era. Our study directly addresses this gap. By employing multivariable logistic regression and external validation in a separate patient cohort, we developed a more robust and generalizable prediction tool. The constructed nomogram quantitatively integrates seven readily accessible clinical parameters—age, cough duration, *M. pneumoniae* infection, atelectasis, lung consolidation, pleural effusion, and pleurisy.

Significantly, our model is calibrated for the contemporary post-pandemic era where the etiological spectrum has broadened. While *M. pneumoniae* remains a key risk factor, our study confirms that other pathogens, including RSV, contribute to PB risk. The nomogram assigns a specific weight to each predictor, translating complex clinical data into a single, visual risk score that estimates the individualized probability of PB. This facilitates early risk stratification, potentially guiding clinicians to prioritize bronchoscopic evaluation for high-risk children, thereby optimizing the timing of the most effective intervention.

The cornerstone of PB management remains the prompt physical removal of casts via bronchoscopy (25). Adjunctive therapies reported include hypertonic saline, bronchodilators, mucolytics (e.g., DNase), fibrinolytic agents (e.g., t-PA, urokinase), and chest physiotherapy (26–28). However, evidence is largely confined to case series, and the optimal medical regimen alongside bronchoscopy is unclear (29). In our practice, bronchoscopic lavage with forceps or basket extraction was central to management. Pre-procedural use of antibiotics and corticosteroids was common, targeting the presumed intense inflammatory drive (30). A major long-term sequela is bronchiolitis obliterans, emphasizing the need for early and complete clearance to prevent permanent airway damage (31).

## 5 Conclusion

In summary, we have developed and validated a novel nomogram for predicting PB risk in children with severe pneumonia. By incorporating a broader range of pathogens and utilizing rigorous multivariable methodology with external validation, this tool addresses the evolving clinical landscape and offers superior applicability compared to previous models. It serves as a practical, evidence-based guide for clinicians to identify children who would benefit most from early diagnostic bronchoscopy, the definitive intervention that can alter the disease course and improve outcomes.

## Data Availability

The raw data supporting the conclusions of the article will be made available by the authors, without undue reservation.
